# Muscular Adaptations to Whole Body Blood Flow Restriction Training and Detraining

**DOI:** 10.3389/fphys.2019.01099

**Published:** 2019-09-10

**Authors:** Christopher R. Brandner, Matthew J. Clarkson, Dawson J. Kidgell, Stuart A. Warmington

**Affiliations:** ^1^Sport Science Department, Aspire Academy for Sports Excellence, Doha, Qatar; ^2^School of Exercise and Nutrition Sciences, Institute for Physical Activity and Nutrition, Deakin University, Burwood, VIC, Australia; ^3^Department of Physiotherapy, School of Primary and Allied Health Care, Monash University, Melbourne, VIC, Australia

**Keywords:** strength training, hypertrophy, vascular occlusion, rehabilitation, BFR, resistance exercise

## Abstract

Resistance training with blood flow restriction is typically performed during single exercises for the lower- or upper-body, which may not replicate *real world* programming. The present study examined the change in muscle strength and mass in a young healthy population during an 8-week whole body resistance training program, as well as monitoring these adaptations following a 4-week detraining period. Thirty-nine participants (27 males, 12 females) were allocated into four groups: blood flow restriction training (BFR-T); moderate-heavy load training (HL-T), light-load training (LL-T) or a non-exercise control (CON). Testing measurements were taken at Baseline, during mid-point of training (week 4), end of training (week 8) and following four weeks of detraining (week 12) and included anthropometrics, body composition, muscle thickness (MTH) at seven sites, and maximal dynamic strength (1RM) for six resistance exercises. Whole body resistance training with BFR significantly improved lower- and upper-body strength (overall; 11% increase in total tonnage), however, this was similar to LL-T (12%), but both groups were lower in comparison with HL-T (21%) and all groups greater than CON. Some markers of body composition (e.g., lean mass) and MTH significantly increased over the course of the 8-week training period, but these were similar across all groups. Following detraining, whole body strength remained significantly elevated for both BFR-T (6%) and HL-T (14%), but only the HL-T group remained higher than all other groups. Overall, whole body resistance training with blood flow restriction was shown to be an effective training mode to increase muscular strength and mass. However, traditional moderate-heavy load resistance training resulted in greater adaptations in muscle strength and mass as well as higher levels of strength maintenance following detraining.

## Introduction

Training using blood flow restriction exercise (BFRE) is gaining popularity among researchers and practitioners such as medical staff, physiotherapists, strength and conditioning coaches and rehabilitation specialists (Patterson and Brandner, 2017). In general, the increase in muscle strength and mass with BFRE is greater than lifting light loads (≤40% 1 repetition maximum [1RM]) without BFR, while often being closely matched to moderate-heavy load (≥65% 1RM) resistance training (Lixandrão et al., 2017). BFR resistance training may provide several advantages over traditional moderate-heavy load resistance training, for example, these muscular adaptations are achieved despite lower relative external loads, produce less muscle damage and thus training frequency can be increased, while muscle hypertrophy has also been shown in as little as 1–2 weeks (Scott et al., 2014). For these reasons, healthy/general populations may be recommended to perform BFRE as part of a training program in order to increase muscular strength, mass, and functional performance or activities of daily living.

BFR has been combined with several different single-joint lower-body (e.g., knee extension, knee flexion, ankle plantarflexion) and upper-body exercises (e.g., elbow flexion, elbow extension) (Patterson and Brandner, 2017), as well as compound multi-joint exercises such as the squat and bench press (Abe et al., 2012). However, the examination of a training program using a limited number of exercises (e.g., one or two) does not reflect typical applied resistance training programs that instead comprise one or more exercises for multiple anatomical regions (American College of Sports Medicine, 2009). Previous studies that have examined multiple resistance exercises with BFR are diverse and include lower-body exercises only (Sumide et al., 2009), upper-body exercises only (Thiebaud et al., 2013), bodyweight exercises (Yokokawa et al., 2008), in-water exercises (Araújo et al., 2015), older adult populations only (Karabulut et al., 2010; Bryk et al., 2016), or clinical cases (Jørgensen et al., 2015; Tennent et al., 2016). With only two of these comparing a BFR training group against traditional moderate-heavy load training (HL-T) in older adults (Karabulut et al., 2010; Bryk et al., 2016). Therefore, a “real world” full-body BFR training program incorporating both upper- and lower-body exercises using traditional weights or weight machines while comparing against a non-BFR control group and a heavy-load resistance exercise group is yet to be investigated.

One other aspect that has been infrequently examined is the effect of detraining on muscular adaptations following BFR training. Detraining is commonly associated with strength loss, muscular atrophy, and reductions in functional capacities in both older adults and clinical populations (Mujika and Padilla, 2000). Physical training in general populations may cease due to injury or illness, time/effort, boredom or fatigue. For athletes, there may be interruptions in training due to competition, travel, offseason rest or taper strategies, or other factors (Mujika and Padilla, 2000). Therefore, while there is an abundance of training studies examining muscular adaptations to BFRE, only few have examined the effects of detraining. Results from these studies have produced mixed results that likely stem from examining different combinations of young adults (Yasuda et al., 2014b, c), older adults (Yasuda et al., 2014a), and the effects of detraining following only lower-body (Yasuda et al., 2014a) or only upper-body training (Yasuda et al., 2014b, c, 2015). From the available literature, muscle strength and some aspects of muscle cross sectional area (CSA) have been found to be maintained for up to 12–24 weeks in older adults, but not differently to non-BFR training or control (non-exercise) group (Yasuda et al., 2014a, 2015). The only study to compare against moderate-HL-T found that after a 3 week detraining period, both the BFR and moderate-heavy load group maintained 1RM bench press strength, but only the moderate-heavy load group maintained pectoralis major and triceps brachii CSA (Yasuda et al., 2014b).

Therefore, the aim of the present study was to investigate the time course adaptations to training (8 weeks) and detraining (4 weeks) in muscle strength and mass to a full-body BFR resistance training program and compare these results to a traditional moderate-heavy load resistance training program and a light-load resistance training (without BFR).

## Materials and Methods

### Participants

Thirty-nine healthy participants (27 males and 12 females; see Table 1 for participant characteristics) volunteered to take part in the study and provided written and informed consent to the experimental procedures. Participants had no known history of peripheral or neurological impairment, cardiovascular, pulmonary, or metabolic disease, musculoskeletal injuries, or self-reported smoking. Additionally, none of the participants had involvement in any kind of resistance training in the previous 2 months. Additionally, participants were asked to refrain from additional exercise and only complete incidental physical activity outside of the study.

**TABLE 1:** Anthropometric characteristics as measured at baseline.

	**BFR-T (*n* = 11**)	**HL-T (*n* = 11)**	**LL-T (*n* = 10)**	**CON (*n* = 7)**
Gender (M, F)	8, 3	7, 4	7, 3	5, 2
Age (years)	23 ± 3	23 ± 3	23 ± 3	24 ± 2
Height (cm)	175.7 ± 12.2	171.5 ± 8.9	177.0 ± 14.0	183.2 ± 8.0
Body mass (kg)	72.5 ± 15.5	71.1 ± 12.0	74.5 ± 21.1	77.5 ± 12.6
BMI (kg.m^–2^)	24 ± 3	24 ± 3	23 ± 4	23 ± 3

*BMI, body mass index; BFR-T, blood flow restriction training; CON, control; HL-T, heavy-load resistance training; LL-T, light-load resistance training.*

### Sample Size

Study sample size was determined by undertaking a power analysis (G^∗^Power v 3.1.9.2). Sample size for the present study was primarily based on lower limb muscle strength, due to a lack of comparative whole body BFR training studies. This was derived from the mean changes observed in previous studies investigating muscular adaptations following knee extension or knee flexion exercise with BFR (Fujita et al., 2008; Karabulut et al., 2010; Kacin and Strazar, 2011). The expected mean changes in strength were approximately 11% for BFR-T, 16% for HL-T, 2% for LL-T, and 0% for CON with a pooled standard deviation of 13%. Target statistical power was set to ≥0.80 to detect significant increases in muscle strength at *P* ≤ 0.05. This analysis suggested that 32 participants would be required across 4 groups, or 8 per group. However, given that this is estimated of single joint training studies, and not whole body exercise, a more conservative 10 per group was targeted.

### Experimental Design

Participants were familiarized with all testing and exercising protocols 3–7 days prior to beginning the study. Testing was conducted prior to training (baseline), at mid-point of training (week 4), end of training (week 8), and following a four-week detraining period (week 12). Therefore, the total duration of the experimental study was 13 weeks. During the training and detraining period, participants were asked to maintain their normal diet and physical activity levels. Following baseline testing, participants were allocated to one of three training groups (total *n* = 32) or a non-training control group (*n* = 7). To minimize covariate imbalance, participants in each group were matched for gender and pre-training knee extension (KE) strength to facilitate equal distribution. However, this method does not necessarily eliminate bias or unknown factors (e.g., other covariates). Testing measurements included anthropometrics (height and body mass), body composition using Dual X-ray Absorptiometry (DXA) and ultrasound to measure muscle thickness (MTH) at seven sites, and maximal dynamic strength (1RM) for six resistance exercises. Testing was completed in this order, with visits lasting for approximately 2–2.5 h. The primary investigator was present to supervise all training sessions throughout the experiment and was assisted by two students of the school.

### Resistance Training Protocols

Participants in the resistance training groups performed 20 training sessions across 8 weeks, three times per week on non-consecutive days. If a participant was unable to attend a scheduled training session, the session was completed elsewhere within the training week. Thus, compliance to the 20 training sessions across 8 weeks was 100%. All training sessions comprised three lower- and three-upper body exercises. Prior to beginning each training session, participants completed a standardized warm up consisting of 5 min cycling on a Monark cycle ergometer (50–100 W). Subsequently, participants began training and completed the exercises in the following order; knee extension (KE), barbell back squat (SQ), calf raise (CR) on a 45° leg press, barbell bench press (BP), seated row (SR), and barbell biceps curl (BC). The loads and repetitions performed for all training groups were different, however, there were some similarities. For all training groups, four sets were performed for KE as an equivalent to the standard four sets performed for BFR training. Only three sets were performed for each of the remaining exercises. Between the three lower- and three upper-body exercises there was a 5-min recovery period, which was approximately the time it would take for the BFR group to deflate and remove lower-body cuffs and then reapply and inflate upper-body cuffs. Loads for training sessions 1–10 were calculated as a percentage of 1RM measured during Baseline strength testing, and sessions 11–20 from the week 4 strength testing session. All repetitions for all resistance exercises for all training groups was monitored by a metronome with a repetition timing of 2 s for the concentric phase and 2 s for the eccentric phase. The total training duration for each training session was approximately 45 min.

### Heavy-Load Training

Participants in the HL-T group (*n* = 11; 3 females) were required to exercise at 70% 1RM. For KE, participants performed four sets of 8–10 repetitions, separated by 1-min rest between sets. Following this, participants completed the additional five resistance exercises, but were only required to complete three sets of 8–10 repetitions. There was a 1-min recovery period between all exercises and sets.

### Light-Load Training

Participants in the LL-T group (*n* = 10; 3 females) were required to exercise at 20% 1RM. For KE, participants performed a total of 30 repetitions in the first set, followed by three sets of 15 repetitions separated by 30 s rest between sets. Following this, participants completed the additional five resistance exercises, but were only required to complete three sets of 15 repetitions.

This set x repetition scheme has been used in previous BFRE studies of similar duration and using multiple exercises (e.g., Weatherholt et al., 2012) and shown to produce increases in muscle strength and size following training. There was a 30 s rest period between sets, and a 1-min recovery period between exercises.

### Blood Flow Restriction Training

Participants in the BFR-T group (*n* = 11; 3 females) were required to perform the same resistance training program as described for LL-T. However, all resistance exercises were completed with BFR at 60% of each individuals limb occlusion pressure (LOP) as previously described from our laboratory (May et al., 2018). Briefly, BFR was applied using an automatic tourniquet system (ATS 3000, Zimmer Inc., OH, United States) connected to inflatable pneumatic cuffs. Cuff widths and lengths for the lower-body (86 cm long, 10.5 cm wide, bladder length 80 cm, bladder width 8 cm) and upper-body (52 cm long, 10.5 cm wide, bladder length 45 cm, bladder width 8 cm) were used throughout the duration of the study. Prior to beginning each training session, participants in the BFR group were fitted with cuffs to the most proximal portion of each thigh in order to perform all three lower-body resistance exercises. The final exercising BFR pressure was set immediately prior to KE and was maintained continuously throughout all three lower-body resistance exercises (approximately 16 min) before the cuffs were deflated. Participants were then given a 5-min recovery time and fitted with cuffs to the most proximal portion of their upper arms. The final exercising BFR pressure was set immediately prior to performing the BP exercise and was maintained continuously throughout all three upper-body resistance exercises (approximately 14 min) before cuff deflation.

To provide individualized restriction pressures, LOP was determined separately for each lower- and upper-body limb for each participant prior to beginning the resistance training protocol (baseline) and at week 4 prior to beginning the second block of training. All measurements were taken in a seated position, as this position was most closely related with the primary outcome exercise (KE), and it has been recommended to measure LOP in the most exercise specific body position (Hughes et al., 2018). With the restriction cuffs in place on the limb, a plethysmograph (LOP Sensor Kit, Zimmer Inc., OH, United States) was applied to the distal process of the second phalange of the foot or hand (second toe/finger) for lower- and upper-body, respectively. Automated measurement of LOP was performed using the inbuilt LOP function (ATS 3000, Zimmer Inc., OH, United States), whereby the restriction cuffs gradually inflated to produce a continuous rise in pressure until tissue blood flow was no longer detected at the measurement site. Measurement of LOP was conducted twice on each limb and were typically within 20 mmHg, whereby the average was then used to set the cuff pressure for exercise. If the measurements were more than 20 mmHg apart on each limb, a third test would be conducted and an average of all three tests would be taken. LOP measures taken at baseline and week 4 of the lower-body (180 ± 7 and 181 ± 8 mmHg, respectively) and upper-body (133 ± 3 and 136 ± 5 mmHg, respectively) were not significantly different. Once LOP was determined, the restriction pressure was set at 60% LOP, equal to 107 ± 5 and 109 ± 5 mmHg for the lower-body and 80 ± 2 and 81 ± 3 mmHg for the upper-body for training sessions 1–10 and 11–20, respectively.

### Control

Participants in the CON group (*n* = 7; 2 females) performed all testing sessions across the duration of the study and were requested not to engage in additional physical activity or exercise outside of their normal daily routine whilst maintaining their normal diet during the study period.

### Anthropometrics

Height and body mass were measured to the nearest 0.5 cm and 0.1 kg, respectively, using a stadiometer (220 portable stadiometer, Seca, Hamburg, Germany) and digital electronic scale (UC-321, A&D Co. Ltd., United States). Body mass index (BMI) was then calculated as; BMI (kg.m^–2^) = Body mass (kg)/Height^2^ (m).

### Muscle Strength and Mass

#### Maximal Strength

Dynamic 1RM testing was performed for the lower-body (knee extension, barbell back squat, calf raise) and upper-body (barbell bench press, seated cable row, barbell biceps curl). Participants completed all 1RM tests through a full range of motion. Before each 1RM test, participants warmed up at 50% of their estimated 1RM. Single repetition lifts were conducted with progressively heavier loads until failure, defined as the final load that could be successful lifted with correct technique where an additional 0.5–5.0 kg could not be successfully lifted. Rest intervals between 1RM attempts were dependent on participant readiness but ranged from 2–5 min, while not more than four to six attempts were completed during any test. During testing, 1RM attempts were alternated between the lower-body and upper-body exercises in order to minimize accumulated fatigue.

#### Body Composition

Dual energy X-ray absorptiometry (DXA; Lunar Prodigy, GE Lunar Corp., Madison, WI, United States) using software version 12.30.008 was used to assess total bone-free lean body mass (LM), total bone-free fat mass (FM), as well as arm-LM, leg-LM, and trunk-LM using a total body scan. Calibration of the DXA was performed on each testing day prior to scanning participants, and scanning procedures were standardized for all participants and done in accordance with recently published best practice protocols for the assessment of whole body composition (Nana et al., 2015). In addition, all analysis of DXA was undertaken by the primary investigator for consistency, who was not blinded to the training group of the participant. The short-term coefficient of variation measured on two consecutive days for repeated measurements of total body lean mass and fat mass in our laboratory ranges from 1.0 to 1.7%. Participants were placed in a supine position with arms placed close to the sides of the body in a neutral position within the 60 cm scanning area on the DXA table. Velcro straps were placed around the ankles to hold the legs together during the scans and prevent any movement.

#### Ultrasound Muscle Thickness

B-mode ultrasonographic evaluation of skeletal MTH was taken at seven sites from the anterior and posterior aspects of the body using a Sonosite ultrasound (Springfield, NJ, United States). All measurements were taken on the participants’ dominant side with subjects lying in supine and prone positions. A 5–15 Hz scanning transducer head was lubricated with transmission gel and placed lightly on the marked area without depressing the dermal surface. Distortion of tissue due to excess compression was eliminated by observing that no movement of the tissue occurred in the real-time ultrasound image. When a clear image was visible on the monitor, the image was captured for immediate analysis. A total of six measurements were taken for each of the anatomical sites (listed below) and the average was used for analysis. The short-term coefficient of variation for repeated measurements on two trials ranged from 1.3 to 6.4%. MTH was determined as the distance between the adipose-muscle interface and muscle-bone interface from the ultrasound image in accordance as per previous protocols (Abe et al., 1994). Briefly, the seven anatomical landmarks of the sites were as follows; *Biceps and triceps:* on the anterior and posterior surface equal to 60% distal between the lateral epicondyle of the humerus and the acromial process of the scapula; *Pectoralis major:* at the clavicular midpoint and between the third and fourth costa; *Quadriceps and hamstring:* on the anterior and posterior surface midway between the lateral condyle of the femur and the greater trochanter; *Gastrocnemius and tibialis anterior:* on the anterior and posterior surface equal to 30% distal of the lateral condyle of the tibia and the lateral malleolus of the fibula. To ensure accuracy of the data across all testing time points, the marking sites were recorded and matched on each testing session.

### Statistical Analysis

All data for measured variables were found to be normally distributed as assessed with a Shapiro-Wilks test (*P* ≤ 0.05). All dependent variables for muscle strength, body composition, and MTH were analyzed for absolute (kg) as well as normalized percentage change from baseline which was calculated as: (*post*−*pre*)/*prex* 100. Total tonnage (TT) was calculated as a sum of all six resistance exercises 1RM at each testing time point, as a reflection of whole body strength. One outlier was removed from the normalized data for CR as the data was 3SD above the mean. A linear mixed model was used to measure main effects for Group (BFR-T, HL-T, LL-T, CON) and Time (Baseline, week 4, week 8, week 12) while also accounting for the small sample size and missing data points. For any Group x Time interactions, a Bonferroni correction was used to determine differences for each dependent variable while accounting for family-wise error. The linear mixed model was performed using SPSS statistical software (v25). The level of significance was set at *P* ≤ 0.05 and all data is presented as mean ± standard deviation (SD) unless stated otherwise.

## Results

There were no significant differences between groups for age, height, body mass, and BMI (Table 1). There was one injury recorded in the HL-T group, with one participant reporting a lower back complaint following training sessions when performing the squat exercise. Therefore, the SQ was removed from their training and all subsequent test sessions whereby their results were not included in the analysis for the SQ or TT. There were no other injuries recorded in any of the other training groups, and no side effects reported for anyone performing BFR exercise (both acute or chronically). One participant in HL-T and one in LL-T were unable to attend the detraining test week and thus this data was not included in the analysis at this time point.

### Training Adaptations

#### Lower-Body Maximal Strength

**FIGURE 1:**
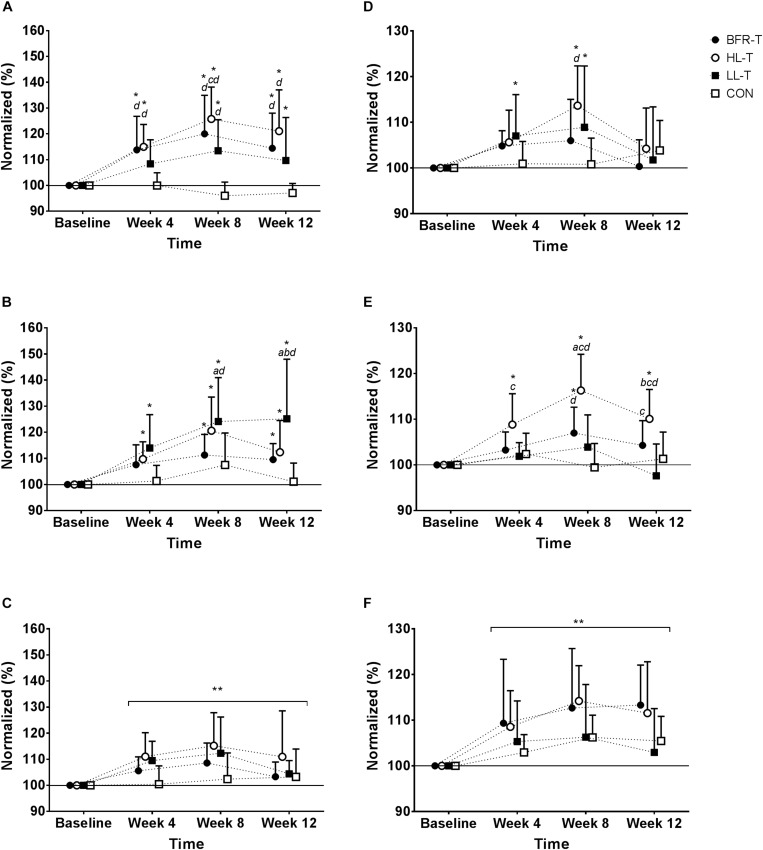
Normalized (%) change in 1RM strength for each exercise. Knee extension **(A)**, Back squat **(B)**, Calf raise **(C)**, Bench press **(D)**, Seated row **(E)**, and Biceps curl **(F)**. ^∗^ indicates significant difference within Group compared with Baseline (*P* ≤ 0.05); ^∗∗^ significant main effect for Time (*P* ≤ 0.05); *a* significantly different to BFR-T; *b* significant different to HL-T; *c* significantly different to LL-T; d significantly different to CON.

Overall, for absolute strength (kg) for KE, SQ, and CR there were no significant main effects for Group (*P* = 0.50–0.94; Table 2). However, there were significant main effects for Time (*P* < 0.0001; Table 2), and significant interactions (Group x Time) for both SQ and KE (*P* < 0.05–0.0001). As such, CON remained similar to baseline across time for all exercises, while in general absolute strength for HL-T, BFR-T, and LL-T increased across time for all exercises (Table 2). Examination of the normalized data (%) elucidated a main effect for both Group (*P* < 0.01) and Time (*P* < 0.0001), and a Group x Time interaction (*P* < 0.0001) for percentage change in KE 1RM strength (Figure 1A). At week 4 and 8 the percentage increase in KE strength was greater in both BFR-T and HL-T compared with CON (*P* < 0.05). The percentage change for HL-T was also significantly greater than LL-T (*P* < 0.0001). KE was only increased at week 8 for LL-T. For SQ normalized data (%), main effects for Group (*P* < 0.01) and Time (*P* ≤ 0.0001) were identified. A Group x Time (*P* < 0.01; Figure 1B) interaction was also detected whereby the percentage increase in SQ 1RM strength was greater for HL-T and LL-T at week 4 (*P* ≤ 0.05), and while the percentage increase was also higher for all groups except CON at week 8, only the percentage increase for LL-T was higher in comparison with BFR-T and CON. There was no main effect for Group, and no Group x Time interaction detected for CR, only a main effect for Time (*P* ≤ 0.0001; Figure 1C).

**TABLE 2:** Absolute (kg) change in 1RM strength.

	**BFR-T**	**HL-T**	**LL-T**	**CON**
**Lower-body strength**				
**Knee extension**				
Baseline	75.58 ± 23.14	79.26 ± 19.61	78.79 ± 20.13	87.47 ± 20.47
Week 4	86.57 ± 30.25^∗^	90.58 ± 21.99^∗^	85.83 ± 24.92	87.87 ± 22.47
Week 8	91.40 ± 32.50^∗^	99.05 ± 24.77^∗^	89.72 ± 26.60^∗^	84.30 ± 19.19
Week 12	89.26 ± 32.00^∗^	96.25 ± 28.91^∗^	87.40 ± 27.59	85.16 ± 20.45
**Back Squat**				
Baseline	82.05 ± 22.20	80.68 ± 19.88	65.00 ± 17.83	85.00 ± 33.17
Week 4	87.27 ± 21.17	86.00 ± 20.55	73.00 ± 17.23^∗^	85.71 ± 33.81
Week 8	90.68 ± 22.19^∗^	93.75 ± 20.92^∗^	79.75 ± 20.50^∗^	88.93 ± 32.17
Week 12	89.55 ± 23.71^∗^	84.16 ± 20.04^∗^	80.83 ± 21.65^∗^	85.36 ± 33.12
**Calf raise**				
Baseline	200.00 ± 50.22	175.00 ± 58.01	167.78 ± 45.49	188.57 ± 52.66
Week 4	211.50 ± 55.18	195.45 ± 55.16^∗^	183.61 ± 51.22^∗^	188.57 ± 52.97
Week 8	217.25 ± 57.96^∗^	206.14 ± 54.26^∗^	185.83 ± 49.31^∗^	190.71 ± 49.87
Week 12	206.67 ± 55.45	197.50 ± 53.35^∗^	173.13 ± 48.62	193.57 ± 57.28
**Upper-body strength**				
**Bench press**				
Baseline	55.32 ± 17.40	55.00 ± 17.54	49.25 ± 17.52	64.29 ± 22.99
Week 4	57.91 ± 18.04	58.18 ± 19.43	52.35 ± 17.95	65.00 ± 23.63
Week 8	58.46 ± 14.27	62.27 ± 20.23^∗^	53.10 ± 18.13^∗^	65.21 ± 24.15
Week 12	55.64 ± 17.53	57.25 ± 21.49	51.11 ± 19.13	66.43 ± 23.36
**Seated row**				
Baseline	52.07 ± 17.04	50.30 ± 12.79	49.68 ± 13.04	58.54 ± 17.38
Week 4	53.55 ± 17.00	54.39 ± 13.20^∗^	50.68 ± 13.47	59.46 ± 15.30
Week 8	54.95 ± 16.13^∗^	58.05 ± 13.72^∗^	51.50 ± 13.27^∗^	58.18 ± 16.99
Week 12	53.77 ± 16.51	55.30 ± 13.53^∗^	49.61 ± 13.86	58.93 ± 15.67
**Biceps curl**				
Baseline	27.82 ± 9.37	27.68 ± 7.03	27.75 ± 10.17	33.36 ± 12.49
Week 4	30.14 ± 9.66^∗^	29.77 ± 6.85^∗^	28.75 ± 9.32	34.29 ± 12.97
Week 8	30.95 ± 9.78^∗^	31.36 ± 7.35^∗^	28.80 ± 8.79	35.50 ± 13.77^∗^
Week 12	31.05 ± 9.47^∗^	30.15 ± 7.95^∗^	29.28 ± 9.08	35.00 ± 13.15

*^∗^ indicates significantly different from Baseline. BFR-T, blood flow restriction training; CON, control; HL-T, heavy-load resistance training; LL-T, light-load resistance training.*

#### Upper-Body Maximal Strength

Overall, absolute strength (kg) for BP, SR, and BC there were no significant main effects for Group (*P* = 0.53–0.70; Table 2). However, there were significant main effects for Time (*P* < 0.0001), as well as significant Group x Time interactions for BP and SR (*P* = 0.05–0.0001). The CON group remained similar to baseline across time for all exercises. In general, upper-body strength increased across time for BP among all groups except CON and SR for HL-T only (Table 2). Examination of the normalized data (%) for percentage change in BP 1RM strength revealed no main effect for Group (*P* = 0.53), but there was for Time (*P* < 0.0001). Additionally, there was a Group x Time interaction (*P* < 0.05; Figure 1D). At week 8, the percentage increase from baseline in BP strength for HL-T and LL-T was significant, but only HL-T was greater than CON. For SR normalized data (%), main effects for Group (*P* < 0.0001) and Time (*P* < 0.0001) were found. A Group x Time (*P* < 0.0001; Figure 1E) interaction was also detected whereby the percentage increase in SR 1RM was increased at week 4 for HL-T only, which was also higher than LL-T. At week 8, the percentage change from baseline was significant for BFR-T and HL-T, and while BFR-T was higher than CON, the percent increase for HL-T was significantly greater than all other groups. There was no main effect for Group, and no Group x Time interaction detected for absolute (kg) or normalized percentage change in BC 1RM, however, there was a main effect for Time (*P* < 0.0001; Figure 1F).

#### Total Tonnage

**FIGURE 2:**
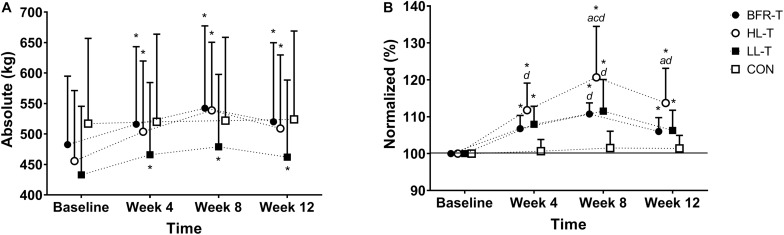
Absolute **(A)** and Normalized **(B)** change in Total Tonnage. ^∗^ indicates significant Group x Time interaction (*P* ≤ 0.0001); *a* significantly different to BFR-T; *b* significant different to HL-T; *c* significantly different to LL-T; d significantly different to CON.

Overall, for absolute TT (kg) there was no main effect for Group (*P* = 0.75). However, there was a main effect for Time (*P* < 0.0001), and a Group x Time interaction (*P* < 0.0001; Figure 2A). There was no change in TT for the CON group throughout training, whereas all other groups increased TT at week 4 and BFR-T and HL-T further increased TT at week 8 (Figure 2A). When examining the normalized data (%) for TT, there was a significant main effect for Group and Time, and a Group x Time interaction (all *P* ≤ 0.0001; Figure 2B). The percentage increase in TT at weeks 4 and 8 was significant for BFR-T, HL-T, and LL-T but not CON. At week 4, the percentage increase was higher for HL-T in comparison with CON. At week 8, the percentage increase was higher for HL-T in comparison with all other groups, and the percentage increase was higher for both BFR-T and LL-T compared with CON.

#### Body Composition

Table 3 displays the body composition (kg) values as represented at Baseline as well as a summary of the main effects and interactions. For access to the full absolute (kg) and normalized (%) body composition changes see Supplementary Tables 1, 2, respectively. When examining the normalized data (%) for body composition, there we no main effects for Group reported, nor any Group x Time interactions. However, there were significant main effects for Time whereby at week 8 all groups showed similar increases in LM, arm-LM, and leg-LM (Table 3 and Supplementary Tables 1, 2).

**TABLE 3:** Body composition and muscle thickness as represented at Baseline.

	**BFR-T**	**HL-T**	**LL-T**	**CON**	**Group**	**Time**	**Group x Time**
**Body composition (kg)**							
Lean mass	52.29 ± 13.06	49.37 ± 10.47	48.54 ± 11.27	56.19 ± 10.82	0.56	**≤0.01**	0.25
Fat mass	16.71 ± 7.27	18.42 ± 6.77	17.17 ± 12.81	17.67 ± 7.81	0.96	0.42	0.48
Arm-LM	6.22 ± 1.86	6.11 ± 1.72	5.83 ± 1.94	7.17 ± 1.73	0.48	**≤0.01**	0.97
Leg-LM	17.67 ± 5.08	16.48 ± 3.59	16.31 ± 4.03	18.45 ± 3.79	0.75	0.07	0.71
Trunk-LM	24.21 ± 6.22	22.57 ± 5.29	22.13 ± 4.57	26.12 ± 4.66	0.38	0.66	0.29
**Muscle thickness (cm)**							
Biceps brachii	2.93 ± 0.51	2.81 ± 0.53	2.92 ± 0.62	3.22 ± 0.65	0.68	**≤0.001**	**≤0.001**
Triceps brachii	3.22 ± 0.83	3.09 ± 0.84	2.82 ± 0.83	3.42 ± 0.99	0.24	**0.02**	0.17
Pectoralis Major	1.56 ± 0.29	1.76 ± 0.30	1.61 ± 0.36	1.89 ± 0.14	0.22	0.53	0.67
Quadriceps	4.28 ± 0.52	4.39 ± 0.82	4.10 ± 0.79	4.14 ± 0.96	0.37	**≤0.001**	**0.03**
Hamstrings	5.56 ± 0.93	5.28 ± 0.85	5.37 ± 1.07	5.46 ± 0.87	0.77	**≤0.001**	0.51
Calf	5.48 ± 1.15	5.35 ± 0.97	5.48 ± 0.91	5.45 ± 0.48	0.73	**≤0.001**	0.35
Tibialis Anterior	3.01 ± 0.34	3.12 ± 0.33	3.21 ± 0.44	3.03 ± 0.37	0.72	0.52	0.50

*Bold values represent significant main effects or interactions for each dependent variable.*

#### Muscle Thickness

Table 3 displays the MTH (cm) values as represented at Baseline as well as a summary of the main effects and interactions. For the full data for absolute (kg) and normalized (%) MTH changes see Supplementary Tables 3, 4. For absolute (cm) MTH, there were no main effects for Group at any measurement site (all *P* > 0.05). However, a significant main effect for Time was detected for all sites (*P* < 0.01) whereby in general MTH increased across the duration of the training program, except for Tibialis Anterior and Pectoralis Major. A Group x Time interaction was detected for Biceps and Quadriceps MTH only (*P* < 0.05). For Biceps MTH, both BFR-T and HL-T were significantly increased at week 4 relative to Baseline, and only HL-T was significantly increased at week 8, with no other changes reported for the other groups. For Quadriceps MTH, BFR-T, HL-T, and LL-T were significantly increased at week 8 relative to baseline.

A similar pattern was found when examining the normalized data (%) for MTH, with main effects for Time reported for all MTH measurement sites (*P* < 0.01) except for Tibialis Anterior and Pectoralis Major. A Group x Time interaction was detected for the normalized change for Biceps brachii and Quadriceps MTH only (*P* < 0.01; Supplementary Table 4). At week 8, HL-T had a significantly greater percentage increase in Biceps MTH compared with baseline, which was also greater than BFR-T, while there were no other significant changes reported for the other groups. The Quadriceps MTH percent change had significantly increased at week 8 for both HL-T and LL-T only, with HL-T also being greater than CON.

### Detraining

Table 2 displays the absolute (kg) strength values for the lower- and upper-body during training and detraining. Following the 4-week detraining period, absolute (kg) KE 1RM strength remained significantly elevated above baseline for BFR-T and HL-T. SQ 1RM strength was also significantly elevated above baseline for BFR-T, HL-T and LL-T. Both CR and SR strength were higher relative to baseline for HL-T only. When examining the normalized data (see Figures 1A–F), KE 1RM strength remained higher at week 12 for all groups except CON. In addition, the KE 1RM strength percentage change for BFR-T and HL-T was higher than CON. The SQ 1RM percentage change was higher at week 12 for all groups except CON, however, only LL-T was higher in comparison with all other groups. For the upper-body, SR 1RM strength remained higher at week 12 for HL-T in comparison with baseline, and was also higher than LL-T and CON, while BFR-T was also significantly higher than LL-T. Following the four-week detraining period, there was also a significant main effect for Time, whereby both CR and BC 1RM strength percentage change remained higher in comparison with Baseline. Overall, the absolute TT remained higher at week 12 relative to baseline for all groups except CON (Figure 2A). The normalized change for TT also remained higher compared with baseline at week 12 for all groups except CON, although HL-T was also higher than both BFR-T and CON (Figure 2B).

Supplementary Tables 1, 2 display the absolute and normalized values for all body composition data, while Supplementary Tables 3, 4 displays the values for MTH data. A main effect for Time was detected for BM, whereby BM was higher at week 12 relative to all other time points (*P* < 0.001). This appeared to be driven by a significant Group x Time interaction for FM whereby FM was also higher at week 12 relative to all other time points. Both HL-T and LL-T had significantly increased FM at week 12 relative to all other time points. At week 12, the percent increase in LM (0.9%) and arm-LM (1.9%) also remained higher relative to baseline levels.

For MTH (cm), a significant main effect for Time remained for Triceps, Quadriceps, Hamstring and Calf with week 12 being greater than Baseline. In addition, the Group x Time interaction remained, with Quadriceps MTH (cm) for HL-T being greater at week 12 relative to Baseline. A similar pattern was detected for MTH (%), with the percent increase for Triceps (5.3%), Quadriceps (4.9%), Hamstring (8.3%) and Calf (6.1%) being higher at week 12 relative to Baseline. In addition, the percent change for Biceps MTH was higher at week 12 for HL-T in comparison with Baseline (7.6%). Finally, the percent change for Quadriceps MTH remained higher at week 12 for HL-T in comparison with Baseline (11.5%).

## Discussion

The present study examined the muscular adaptations to an 8-week whole body resistance training program both with and without BFR, and the effects of a 4-week detraining period. To our knowledge, this is the first study to examine adaptations in muscle strength and mass to a whole body resistance training program with BFR and compare these to moderate-heavy load and light-load non-BFR training in a young adult population. The major findings showed that muscle strength and mass increased for BFR-T to different degrees for each exercise/muscle group, which was similar to LL-T, and overall the increase in whole body strength appeared to be higher for HL-T in comparison with all other groups. The increase in muscle mass was similar for all training groups. Furthermore, following 4-weeks of detraining, whole body strength increases were maintained following all groups other than CON in the present study, but only the HL-T was significantly greater than the other groups. These results suggest that BFR training is an effective mode of exercise to improve muscle strength and mass when undertaken as part of a whole body program (i.e., incorporating three upper-body and three lower-body exercises), with these improvements being similar to traditional moderate-heavy load or light-load training (LL-T) without BFR.

### Training Adaptations

On closer examination of the individual exercise changes in absolute 1RM strength, BFR-T produced significant increases in 5 of 6 exercises, the same as LL-T, while all 6 exercises improved for HL-T (see Table 2). However, examination of percent change may be more prudent to examine throughout the following discussion as it allows for a clear and relative depiction of the changes for each group.

Over the first four-week training period, the increase in KE strength as one of the main outcome measures was significantly greater for BFR-T (14%) and HL-T (15%) when compared with CON, while these were not different to LL-T despite the differing magnitude of change (8%). However, Quadriceps MTH was only increased for HL-T and not any other group. While this contrasts with previous studies that report quite rapid gains in muscle mass with BFR training [e.g., within 1–2 weeks; (Loenneke et al., 2011; Scott et al., 2014)], these studies have used greater training frequencies (1–2 times per day) and performed training to muscular failure. Continuation of the training program for a further four weeks resulted in all three groups increasing KE 1RM strength and while the percentage change for all groups was significant, only the HL-T group was greater than both LL-T and CON, while BFR-T was greater than CON but similar to LL-T.

In contrast to the present study, much of the previous literature comparing the change in absolute/normalized 1RM strength and muscle mass show greater adaptations following BFR in comparison with load matched controls (Loenneke et al., 2011). However, the typical focus of BFR literature is on “single” exercises or “dual” exercises for the lower- (Sumide et al., 2009) or upper-body (Thiebaud et al., 2013), with very few attempting under significantly variable conditions, or attempting to compare with traditional training modes (Karabulut et al., 2010; Bryk et al., 2016). While important in establishing the likely outcomes for BFR exercise training, resistance training programs should contain several exercises for both the lower- and upper-body in order maximize the gains in muscular adaptations (American College of Sports Medicine, 2009). Therefore, a major novelty of the present study was that BFR was performed during a whole body resistance training program in a young adult population. This effectively increased the total volume of work (sets x repetitions) performed across a large number of muscle groups, some of which may have been involved in more than one exercise (e.g., increased quadriceps muscle involvement with the combination of KE and SQ). Given there exists a strong possibility for a dose-response relationship between muscular adaptations and BFR resistance training volume, at least up to a certain volume (Martín-Hernández et al., 2013) it is probable that the similarities in strength and mass between the BFR-T and LL-T training groups can be explained by the increased total volume of work performed. Additionally, previous studies have shown that longer training durations (≥8 weeks) produces similar improvements in muscle strength and mass for LL-T with and without BFR (Fitschen et al., 2013; Barcelos et al., 2015) which agrees with the results of the present study.

Examination of the individual exercise response to the present training program as a whole is difficult, given that each exercise increased differently between and within-groups across the 8-week training program. Therefore, we attempted to summarize the data by calculating whole body strength via TT. Overall, the percentage change in TT was highest for HL-T following the 8-week training program (21%). Further, the increase in TT was similar for BFR-T (11%) and LL-T (12%), with all groups stronger than CON (1%). In summary, whole body strength increased following training in the following manner: HL-T > BFR-T = LL-T > CON. Previous studies have observed similar adaptations in muscle strength between BFRE and moderate-HL-T (Takarada et al., 2000) while others have demonstrated lower responses for BFRE in comparison with moderate-HL-T (Lixandrão et al., 2015). The differences in results between studies could be explained by different populations, exercise selection and training protocols, and the BFR methodology being used between studies. However, a recent meta-analysis comparing the two modes of resistance training found that traditional moderate-heavy load resistance training produced a 7% advantage in strength when compared with BFR (Lixandrão et al., 2017), which is in line with the results from the present study. Interestingly, the same meta-analysis also found that muscle mass increased similarly between the two modes of exercise, which was generally observed in the results from the present study. To highlight this, there were no between-group differences across the training program for lean mass, arm- or leg-lean mass, or any of the seven MTH sites.

Based on the results of the present study as well as data from previous literature (Loenneke et al., 2011; Lixandrão et al., 2017), it appears that LL-T both with and without BFR, was less effective for the development of muscle strength in comparison with traditional moderate-HL-T and should thus question the training protocols used in order to explain our results. BFR pressures were individualized for each participant, with pressures equal to 60% LOP. While the “optimal” BFR pressure to induce maximal adaptations in muscle strength or mass is not known, 40–80% of the maximal limb/arterial occlusion pressures have been recommended previously (Patterson and Brandner, 2017), while Counts et al. (2015) recently showed similar adaptations in muscle strength and endurance following 8 weeks of BFR with either 40 or 90% of the maximal occlusion pressure. Some individuals in the present study did not increase their 1RM strength in one or more of the exercises at the end of week 4, and thus a progressive overload stimulus was not applied for the next four-week training period. For the BFR-T and LL-T groups, if the training loads (kg) lifted progressed in each training period, or each week were progressively overloaded (e.g., 20, 30, then 40% 1RM), then perhaps greater gains in both muscle strength and mass may have been observed and this would likely reflect what would occur in a *real world* training program or rehabilitation setting. BFRE has previously been shown to be effective at increasing muscular endurance and hypertrophy with loads as low as 15% MVIC (Kacin and Strazar, 2011), but this same load is likely not sufficient for maximizing 1RM strength without training at a load closer to maximal intensity (Jessee et al., 2018). Although in some instances, such improvements have been shown to occur between loads ranging from 20 to 50% 1RM (Barcelos et al., 2015). Based on this information, while it is likely that participants were training at a sufficient BFR pressure and training load throughout the present study, there may be some degree of load-specificity required to maximize strength adaptations. Consequently, low-load whole body resistance training with BFR may not be the best way to apply this technique in younger adults if the aim of resistance training is to improve muscle strength. BFRE may be better suited for populations who are not contraindicated to lifting moderate-heavy loads (i.e., young, healthy adults, athletic populations) as a supplement to their regular training at the end of their workouts (Yamanaka et al., 2012; Luebbers et al., 2014). Another alternative would be to combine traditional moderate-HL-T with BFRE throughout a periodized training week, a method which has been shown to be more effective than BFRE alone (Yasuda et al., 2010). While the results of this study support the use of moderate-HL-T to develop muscle strength and mass in young healthy untrained populations, it may be expected that for individuals unable to lift heavy-loads (e.g., the elderly, following musculoskeletal injury, or where muscle atrophy and weakness occur due to the effects of inactivity or disease), that a multi-exercise program using LL-T with or without BFR may also be effective at increasing muscle strength during individual exercises and muscle mass at various anatomical sites.

### Effects of Detraining

Results from the present study show that following the four-week detraining period, both KE and SQ strength remained higher in comparison with baseline for BFR-T, while there was either no change in strength for all other resistance exercises during the training period, or they returned to baseline levels. Previously, both Yasuda et al. (2015) and Yasuda et al. (2014b) demonstrated that lower body strength can be maintained for longer detraining periods (12–24 weeks) following BFR training, however, those studies were performed in older adults (≥65 years). Therefore, to our knowledge this was the first study to observe that lower body strength can be maintained for short periods of detraining in a young healthy population following whole body BFR training. When the strength data was combined to calculate the TT, a metric of whole body strength, 1RM strength for BFR-T remained higher at week 12 when compared with baseline. However, it is important to note that while KE, SQ, and TT strength remained elevated, these were not different to the other groups. Overall, only the HL-T group maintained a training-related increase in whole body TT strength relative to baseline levels, which was also greater than all other groups. Similarly, the increase in strength for SR and BC observed in the present study following BFR-T was only maintained after detraining for BC, and this was also not different to the other groups. Previously, Yasuda et al. (2014b) had shown that following six weeks of bench press training with BFR in young males (22–27 years), not only had 1RM strength significantly increased by 4.3%, but this remained elevated by 4.9% following 3 weeks of detraining. Upon closer examination, the BFR-T group in the present study produced a non-significant increase in BP 1RM similarly to Yasuda et al. (2014b) by 6% following training, so it is unknown why these adaptations were not maintained in the present cohort except that we measured an additional one week of detraining.

Of the previous studies reporting the effects of detraining following BFR, while strength has been shown to be maintained, muscle mass appears to return to baseline levels despite improvements during the training program (Yasuda et al., 2014a, b). This effect was also apparent in the present study with no significant Group x Time interactions detected for BFR-T. However, there were significant main effects for Time whereby LM (0.9%), Arm-LM (1.9%), Triceps MTH (5.3%), Quadriceps MTH (4.9%), Hamstring MTH (8.3%) and Calf MTH (6.1%) all remained elevated above baseline levels. Importantly, these percentage increases appear not to be driven by the CON group, and collectively, despite the lower total volume load lifted by the BFR-T and LL-T groups in comparison to HL-T, all training groups were able to maintain some improvement in muscle mass throughout the detraining period. It should also be noted that the Quadriceps MTH for HL-T remained 12% higher at week 12, which was significantly higher than all other groups. Therefore, it is probable that changes in muscle architecture were also responsible for strength maintenance for HL-T during detraining, similar to previous literature (Narici et al., 1989; Hakkinen et al., 2000). Overall, given that both TT and several body composition and MTH measurements remained significantly elevated following detraining for HL-T, which were higher than all other groups, it appears that traditional moderate-heavy load (70% 1RM) training had the greatest effect on strength and mass maintenance across the four-week detraining period.

### Limitations

The sample size in the present study, while satisfying the sample size calculations, is objectively a small sample size given the number of groups (4), testing time points (4), and multiple measures of muscle strength and mass. However, the use of linear mixed models for our statistical analysis accounts for smaller sample sizes well and also overcomes the presence of a small number of missing data points. In addition, whilst we attempted to recruit male and female participants, it was not the purpose of the current study to compare muscular adaptations between genders. Thus, given the disproportionate sample of males (27) to females (12) we attempted to balance genders across training groups and statistical comparisons were not made. We did not monitor physical exercise or nutrition outside of the study, although participants were aware that no additional resistance training should take place throughout the study period, especially including the detraining period (including CON). As mentioned in the discussion, we did not control for a learning effect in our strength measurements. Given the recruitment of participants were novice lifters, it is possible that an initial familiarization (i.e., multiple weeks of training prior to testing) may have diminished any potential learning effect, however, this was not done in the current study.

## Conclusion

The present study examined the change in muscle strength and mass in a young healthy population during an 8-week whole body resistance training program, as well as monitoring these adaptations following a 4-week detraining period. The results showed that whole body resistance training with BFR significantly improved lower-body and upper-body strength (overall; 11% increase in TT), however, this was similar to LL-T (12% increase in TT), but both groups were lower in comparison with traditional moderate-HL-T (21% increase in TT) and all groups greater than CON. Some markers of body composition (e.g., lean mass) and MTH significantly increased over the course of the 8-week training period, but these were similar across all groups. Finally, whole body strength remained significantly elevated following the four-week detraining period for BFR-T (6%), HL-T (14%), and LL-T (6%) but only the HL-T group remained higher than any of the other groups. Overall, a whole body resistance training program with BFR was shown to be an effective training mode to increase muscular strength during training and remain elevated following four weeks of detraining. However, the present study appears to show that resistance training with moderate-heavy loads (70% 1RM) results in greater adaptations in strength and muscle mass as well as higher levels of strength maintenance following detraining.

## Ethics Statement

This study was approved by the Human Research Ethics Committee of Deakin University (project identification: HREC 2011-228), and all experiments were conducted according to the standards established by the Declaration of Helsinki.

## Author Contributions

CB, DK, and SW conceived and designed the research. CB conducted the experiments. CB, MC, and SW analyzed the data. All authors wrote, edited, and approved the manuscript.

## Conflict of Interest Statement

The authors declare that the research was conducted in the absence of any commercial or financial relationships that could be construed as a potential conflict of interest.
