# Supplementary material for: Muscular Adaptations to Whole Body Blood Flow Restriction Training and Detraining
Source: Front Physiol. 2019 Sep 10;10:1099. doi: 10.3389/fphys.2019.01099 (PMC6746941; doi:10.3389/fphys.2019.01099)
Supplement: Supplementary file 1 [file Table_1.DOCX]

**Supplementary Table 1.** Absolute (kg) change in body composition. BFR-T, blood flow restriction training; CON, control; HL-T, heavy-load resistance training; LL-T, light-load resistance training.

|  |  | **Baseline** | | | **Week 4** | | | **Week 8** | | | **Week 12** | | |
| --- | --- | --- | --- | --- | --- | --- | --- | --- | --- | --- | --- | --- | --- |
| **Lean mass** |  | **Ave** | **SD** | **Sig.** | **Ave** | **SD** | **Sig.** | **Ave** | **SD** | **Sig.** | **Ave** | **SD** | **Sig.** |
|  | **BFR-T** | 52.3 | 13.0 |  | 52.3 | 13.2 |  | 52.6 | 12.7 | # | 52.5 | 13.4 | # |
|  | **HL-T** | 49.4 | 10.5 |  | 49.8 | 10.5 |  | 50.4 | 10.4 | #* | 49.1 | 10.8 | # |
|  | **LL-T** | 48.5 | 11.3 |  | 49.1 | 11.2 |  | 48.9 | 11.0 | # | 49.2 | 12.4 | # |
|  | **CON** | 56.2 | 10.8 |  | 56.5 | 10.4 |  | 57.2 | 11.2 | #* | 56.6 | 10.5 | # |
| **Fat mass** |  | **Ave** | **SD** | **Sig.** | **Ave** | **SD** | **Sig.** | **Ave** | **SD** | **Sig.** | **Ave** | **SD** | **Sig.** |
|  | **BFR-T** | 16.7 | 7.3 |  | 16.5 | 6.9 |  | 16.3 | 6.8 |  | 16.2 | 6.7 |  |
|  | **HL-T** | 18.4 | 6.8 |  | 18.6 | 7.0 |  | 18.4 | 7.1 |  | 20.1 | 6.0 |  |
|  | **LL-T** | 17.2 | 12.8 |  | 17.3 | 13.0 |  | 17.9 | 13.6 |  | 19.8 | 14.0 |  |
|  | **CON** | 17.7 | 7.8 |  | 17.5 | 8.0 |  | 17.4 | 7.5 |  | 17.9 | 7.1 |  |
| **Arm lean mass** |  | **Ave** | **SD** | **Sig.** | **Ave** | **SD** | **Sig.** | **Ave** | **SD** | **Sig.** | **Ave** | **SD** | **Sig.** |
|  | **BFR-T** | 6.2 | 1.9 |  | 6.3 | 2.0 |  | 6.3 | 1.9 | # | 6.3 | 1.9 | # |
|  | **HL-T** | 6.1 | 1.7 |  | 6.2 | 1.6 |  | 6.3 | 1.7 | # | 6.1 | 1.7 | # |
|  | **LL-T** | 5.8 | 1.9 |  | 5.9 | 1.9 |  | 5.9 | 1.9 | # | 6.0 | 2.1 | # |
|  | **CON** | 7.2 | 1.7 |  | 7.3 | 1.8 |  | 7.3 | 1.8 | # | 7.3 | 1.8 | # |
| **Leg lean mass** |  | **Ave** | **SD** | **Sig.** | **Ave** | **SD** | **Sig.** | **Ave** | **SD** | **Sig.** | **Ave** | **SD** | **Sig.** |
|  | **BFR-T** | 17.7 | 5.1 |  | 17.9 | 5.0 |  | 17.8 | 5.1 |  | 17.6 | 5.0 |  |
|  | **HL-T** | 16.5 | 3.6 |  | 16.8 | 3.5 |  | 16.7 | 3.5 |  | 16.3 | 3.3 |  |
|  | **LL-T** | 16.3 | 4.0 |  | 16.8 | 3.9 |  | 16.8 | 3.9 |  | 16.4 | 4.4 |  |
|  | **CON** | 18.5 | 3.8 |  | 18.3 | 3.7 |  | 18.5 | 3.9 |  | 18.5 | 3.9 |  |
| **Trunk lean mass** |  | **Ave** | **SD** | **Sig.** | **Ave** | **SD** | **Sig.** | **Ave** | **SD** | **Sig.** | **Ave** | **SD** | **Sig.** |
|  | **BFR-T** | 24.2 | 6.2 |  | 23.8 | 5.9 |  | 23.9 | 5.3 |  | 23.8 | 6.1 |  |
|  | **HL-T** | 22.6 | 5.3 |  | 22.6 | 5.3 |  | 23.0 | 4.8 |  | 22.2 | 5.2 |  |
|  | **LL-T** | 22.1 | 4.6 |  | 22.0 | 5.2 |  | 21.8 | 4.6 |  | 22.5 | 5.4 |  |
|  | **CON** | 26.1 | 4.7 |  | 26.3 | 4.4 |  | 26.4 | 4.7 |  | 26.7 | 4.8 |  |

* indicates significant (*P* ≤ 0.05) difference from Baseline; # main effect for Time vs Baseline (*P* ≤ 0.05)
