# Supplementary material for: Muscular Adaptations to Whole Body Blood Flow Restriction Training and Detraining
Source: Front Physiol. 2019 Sep 10;10:1099. doi: 10.3389/fphys.2019.01099 (PMC6746941; doi:10.3389/fphys.2019.01099)
Supplement: Supplementary file 2 [file Table_2.DOCX]

**Supplementary Table 2.** Normalized (%) change in body composition. BFR-T, blood flow restriction training; CON, control; HL-T, heavy-load resistance training; LL-T, light-load resistance training.

|  |  | **Baseline** | | | **Week 4** | | | **Week 8** | | | **Week 12** | | |
| --- | --- | --- | --- | --- | --- | --- | --- | --- | --- | --- | --- | --- | --- |
| **Lean mass** |  | **Ave** | **SD** | **Sig.** | **Ave** | **SD** | **Sig.** | **Ave** | **SD** | **Sig.** | **Ave** | **SD** | **Sig.** |
|  | **BFR-T** | 100.00 | - |  | 99.95 | 1.60 |  | 100.69 | 1.44 | # | 100.28 | 1.96 | # |
|  | **HL-T** | 100.00 | - |  | 100.90 | 2.07 |  | 102.27 | 2.05 | #* | 100.80 | 1.48 | # |
|  | **LL-T** | 100.00 | - |  | 101.33 | 1.85 |  | 100.85 | 1.38 | # | 101.61 | 2.05 | # |
|  | **CON** | 100.00 | - |  | 100.63 | 1.17 |  | 101.77 | 1.47 | # | 100.86 | 1.24 | # |
| **Fat mass** |  | **Ave** | **SD** | **Sig.** | **Ave** | **SD** | **Sig.** | **Ave** | **SD** | **Sig.** | **Ave** | **SD** | **Sig.** |
|  | **BFR-T** | 100.00 | - |  | 99.54 | 8.32 |  | 98.03 | 11.06 |  | 98.76 | 16.61 |  |
|  | **HL-T** | 100.00 | - |  | 100.24 | 5.36 |  | 98.94 | 8.49 |  | 102.50 | 8.88 |  |
|  | **LL-T** | 100.00 | - |  | 100.68 | 5.18 |  | 103.81 | 5.20 |  | 104.87 | 8.32 |  |
|  | **CON** | 100.00 | - |  | 98.15 | 3.89 |  | 99.04 | 5.37 |  | 103.43 | 9.41 |  |
| **Arm lean mass** |  | **Ave** | **SD** | **Sig.** | **Ave** | **SD** | **Sig.** | **Ave** | **SD** | **Sig.** | **Ave** | **SD** | **Sig.** |
|  | **BFR-T** | 100.00 | - |  | 100.57 | 3.07 |  | 101.76 | 4.39 | # | 101.59 | 2.97 | # |
|  | **HL-T** | 100.00 | - |  | 101.70 | 5.25 |  | 102.79 | 2.82 | #* | 103.41 | 1.71 | #* |
|  | **LL-T** | 100.00 | - |  | 100.64 | 3.59 |  | 101.71 | 3.64 | # | 101.09 | 4.50 | # |
|  | **CON** | 100.00 | - |  | 101.93 | 1.11 |  | 102.06 | 2.83 | # | 101.56 | 1.18 | # |
| **Leg lean mass** |  | **Ave** | **SD** | **Sig.** | **Ave** | **SD** | **Sig.** | **Ave** | **SD** | **Sig.** | **Ave** | **SD** | **Sig.** |
|  | **BFR-T** | 100.00 | - |  | 101.64 | 3.18 |  | 100.94 | 4.34 |  | 100.05 | 2.95 |  |
|  | **HL-T** | 100.00 | - |  | 101.89 | 3.20 |  | 101.43 | 4.02 |  | 101.18 | 3.58 |  |
|  | **LL-T** | 100.00 | - |  | 103.18 | 4.70 |  | 103.66 | 4.51 |  | 100.61 | 3.02 |  |
|  | **CON** | 100.00 | - |  | 99.32 | 1.32 |  | 100.36 | 2.28 |  | 100.20 | 3.22 |  |
| **Trunk lean mass** |  | **Ave** | **SD** | **Sig.** | **Ave** | **SD** | **Sig.** | **Ave** | **SD** | **Sig.** | **Ave** | **SD** | **Sig.** |
|  | **BFR-T** | 100.00 | - |  | 98.62 | 4.07 |  | 99.48 | 4.35 |  | 98.51 | 3.91 |  |
|  | **HL-T** | 100.00 | - |  | 100.19 | 4.20 |  | 102.81 | 6.74 |  | 99.37 | 4.65 |  |
|  | **LL-T** | 100.00 | - |  | 98.87 | 4.80 |  | 98.69 | 3.39 |  | 102.33 | 4.56 |  |
|  | **CON** | 100.00 | - |  | 100.84 | 2.72 |  | 101.37 | 3.53 |  | 102.38 | 3.55 |  |

* indicates significant difference from Baseline (*P* ≤ 0.05); # main effect for Time vs Baseline (*P* ≤ 0.05).
