# Supplementary material for: Muscular Adaptations to Whole Body Blood Flow Restriction Training and Detraining
Source: Front Physiol. 2019 Sep 10;10:1099. doi: 10.3389/fphys.2019.01099 (PMC6746941; doi:10.3389/fphys.2019.01099)
Supplement: Supplementary file 3 [file Table_3.DOCX]

**Supplementary Table 3.** Absolute (cm) change in muscle thickness (MTH). BFR-T, blood flow restriction training; CON, control; HL-T, heavy-load resistance training; LL-T, light-load resistance training.

|  |  | **Baseline** | | | | **Week 4** | | | | **Week 8** | | | | **Week 12** | | | |
| --- | --- | --- | --- | --- | --- | --- | --- | --- | --- | --- | --- | --- | --- | --- | --- | --- | --- |
| **Biceps brachii MTH** |  | **Ave** | **SD** | **Sig.** | **Ave** | | **SD** | **Sig.** | **Ave** | | **SD** | **Sig.** | **Ave** | | **SD** | **Sig.** |  |
|  | **BFR-T** | 2.94 | 0.52 |  | 3.06 | | 0.47 | *‡# | 2.94 | | 0.51 | ‡# | 2.87 | | 0.59 |  |  |
|  | **HL-T** | 2.81 | 0.54 |  | 2.95 | | 0.57 | *# | 3.08 | | 0.48 | *‡# | 2.95 | | 0.52 | * |  |
|  | **LL-T** | 2.93 | 0.63 |  | 2.99 | | 0.63 | # | 3.05 | | 0.61 | ‡# | 3.04 | | 0.59 |  |  |
|  | **CON** | 3.22 | 0.65 |  | 3.26 | | 0.61 | # | 3.31 | | 0.60 | ‡# | 3.21 | | 0.66 |  |  |
| **Triceps brachii MTH** |  | **Ave** | **SD** | **Sig.** | **Ave** | | **SD** | **Sig.** | **Ave** | | **SD** | **Sig.** | **Ave** | | **SD** | **Sig.** |  |
|  | **BFR-T** | 3.22 | 0.83 |  | 3.13 | | 0.61 |  | 3.43 | | 0.75 | ^ | 3.35 | | 0.70 | ^ |  |
|  | **HL-T** | 3.10 | 0.85 |  | 3.26 | | 0.77 |  | 3.45 | | 0.73 | * | 3.45 | | 0.55 |  |  |
|  | **LL-T** | 2.82 | 0.59 |  | 2.79 | | 0.59 |  | 2.94 | | 0.45 |  | 2.90 | | 0.64 |  |  |
|  | **CON** | 3.43 | 1.00 |  | 3.49 | | 0.99 |  | 3.56 | | 1.03 |  | 3.66 | | 0.85 |  |  |
| **Pectoralis Major MTH** |  | **Ave** | **SD** | **Sig.** | **Ave** | | **SD** | **Sig.** | **Ave** | | **SD** | **Sig.** | **Ave** | | **SD** | **Sig.** |  |
|  | **BFR-T** | 1.56 | 0.30 |  | 1.62 | | 0.32 |  | 1.75 | | 0.37 | *# | 1.62 | | 0.27 |  |  |
|  | **HL-T** | 1.77 | 0.30 |  | 1.86 | | 0.31 |  | 1.99 | | 0.30 | # | 1.88 | | 0.24 |  |  |
|  | **LL-T** | 1.61 | 0.36 |  | 1.65 | | 0.24 |  | 1.71 | | 0.34 | # | 1.67 | | 0.28 |  |  |
|  | **CON** | 1.89 | 0.14 |  | 1.90 | | 0.19 |  | 1.85 | | 0.22 | # | 1.89 | | 0.18 |  |  |
| **Quadriceps MTH** |  | **Ave** | **SD** | **Sig.** | **Ave** | | **SD** | **Sig.** | **Ave** | | **SD** | **Sig.** | **Ave** | | **SD** | **Sig.** |  |
|  | **BFR-T** | 4.28 | 0.53 |  | 4.44 | | 0.51 |  | 4.57 | | 0.62 | #†* | 4.40 | | 0.67 | # |  |
|  | **HL-T** | 4.39 | 0.83 |  | 4.58 | | 0.75 |  | 4.95 | | 0.59 | #†*^ | 4.85 | | 0.57 | #* |  |
|  | **LL-T** | 4.10 | 0.79 |  | 4.37 | | 0.83 |  | 4.48 | | 0.77 | #†* | 4.33 | | 0.89 | # |  |
|  | **CON** | 4.14 | 0.97 |  | 4.03 | | 0.75 |  | 4.12 | | 0.87 | #† | 4.11 | | 0.86 | # |  |
| **Hamstrings MTH** |  | **Ave** | **SD** | **Sig.** | **Ave** | | **SD** | **Sig.** | **Ave** | | **SD** | **Sig.** | **Ave** | | **SD** | **Sig.** |  |
|  | **BFR-T** | 5.57 | 0.94 |  | 5.83 | | 0.81 |  | 5.97 | | 0.96 | #†* | 6.12 | | 0.75 | # |  |
|  | **HL-T** | 5.28 | 0.85 |  | 5.50 | | 0.79 |  | 6.01 | | 0.68 | #†* | 5.98 | | 0.60 | #* |  |
|  | **LL-T** | 5.37 | 1.08 |  | 5.37 | | 0.96 |  | 5.83 | | 0.83 | #† | 5.73 | | 0.96 | # |  |
|  | **CON** | 5.47 | 0.87 |  | 5.59 | | 0.79 |  | 5.66 | | 0.86 | #† | 5.56 | | 0.90 | # |  |
| **Calf MTH** |  | **Ave** | **SD** | **Sig.** | **Ave** | | **SD** | **Sig.** | **Ave** | | **SD** | **Sig.** | **Ave** | | **SD** | **Sig.** |  |
|  | **BFR-T** | 5.49 | 1.15 |  | 5.85 | | 0.86 |  | 6.07 | | 0.84 | #* | 5.92 | | 0.87 | #* |  |
|  | **HL-T** | 5.35 | 0.97 |  | 5.52 | | 0.92 |  | 5.82 | | 0.73 | #* | 5.76 | | 0.78 | #* |  |
|  | **LL-T** | 5.49 | 0.92 |  | 5.56 | | 0.83 |  | 5.81 | | 0.87 | # | 5.71 | | 0.86 | # |  |
|  | **CON** | 5.46 | 0.49 |  | 5.36 | | 0.61 |  | 5.41 | | 0.63 | # | 5.40 | | 0.50 | # |  |
| **Tibialis Anterior MTH** |  | **Ave** | **SD** | **Sig.** | **Ave** | | **SD** | **Sig.** | **Ave** | | **SD** | **Sig.** | **Ave** | | **SD** | **Sig.** |  |
|  | **BFR-T** | 3.01 | 0.35 |  | 3.05 | | 0.45 |  | 3.10 | | 0.47 |  | 3.08 | | 0.34 |  |  |
|  | **HL-T** | 3.13 | 0.33 |  | 3.21 | | 0.36 |  | 3.16 | | 0.37 |  | 3.18 | | 0.37 |  |  |
|  | **LL-T** | 3.21 | 0.44 |  | 3.23 | | 0.48 |  | 3.21 | | 0.41 |  | 3.21 | | 0.46 |  |  |
|  | **CON** | 3.03 | 0.37 |  | 3.03 | | 0.35 |  | 3.00 | | 0.36 |  | 3.05 | | 0.32 |  |  |

* indicates significant difference from Baseline (*P* ≤ 0.05); ^ indicates significant difference from 4 weeks (*P* ≤ 0.05); # main effect for Time vs Baseline (*P* ≤ 0.05); † main effect for Time vs 4 weeks (*P* ≤ 0.05); ‡ main effect for Time vs Week 12 (*P* ≤ 0.05).
