# Supplementary material for: Muscular Adaptations to Whole Body Blood Flow Restriction Training and Detraining
Source: Front Physiol. 2019 Sep 10;10:1099. doi: 10.3389/fphys.2019.01099 (PMC6746941; doi:10.3389/fphys.2019.01099)
Supplement: Supplementary file 4 [file Table_4.DOCX]

**Supplementary Table 4.** Normalized (%) change in muscle thickness (MTH). BFR-T, blood flow restriction training; CON, control; HL-T, heavy-load resistance training; LL-T, light-load resistance training.

|  |  | **Baseline** | | | **Week 4** | | | **Week 8** | | | **Week 12** | | |
| --- | --- | --- | --- | --- | --- | --- | --- | --- | --- | --- | --- | --- | --- |
| **Biceps brachii MTH** |  | **Ave** | **SD** | **Sig.** | **Ave** | **SD** | **Sig.** | **Ave** | **SD** | **Sig.** | **Ave** | **SD** | **Sig.** |
|  | **BFR-T ^$^** | 100.00 |  |  | 105.07 | 6.60 | # | 100.46 | 4.75 | #*b* | 97.39 | 4.87 | ^*b* |
|  | **HL-T** | 100.00 |  |  | 105.29 | 6.04 | #* | 110.59 | 9.04 | #*^*ad* | 107.58 | 8.83 | **ad* |
|  | **LL-T** | 100.00 |  |  | 102.48 | 6.78 | # | 104.69 | 6.67 | # | 104.15 | 5.90 |  |
|  | **CON** | 100.00 |  |  | 101.60 | 3.74 | # | 103.36 | 4.19 | #*b* | 99.89 | 5.21 | *b* |
| **Triceps brachii MTH** |  | **Ave** | **SD** | **Sig.** | **Ave** | **SD** | **Sig.** | **Ave** | **SD** | **Sig.** | **Ave** | **SD** | **Sig.** |
|  | **BFR-T** | 100.00 |  |  | 99.62 | 12.62 |  | 108.24 | 12.42 | # | 108.50 | 13.17 | # |
|  | **HL-T** | 100.00 |  |  | 107.75 | 20.73 |  | 113.99 | 16.47 | #**c* | 109.75 | 16.56 | #* |
|  | **LL-T** | 100.00 |  |  | 98.79 | 8.24 |  | 100.94 | 7.88 | #*b* | 99.25 | 11.16 | # |
|  | **CON** | 100.00 |  |  | 101.97 | 7.75 |  | 100.04 | 7.75 | # | 103.86 | 5.55 | # |
| **Pectoralis Major MTH** |  | **Ave** | **SD** | **Sig.** | **Ave** | **SD** | **Sig.** | **Ave** | **SD** | **Sig.** | **Ave** | **SD** | **Sig.** |
|  | **BFR-T** | 100.00 |  |  | 103.78 | 3.77 |  | 113.16 | 16.69 | #* | 105.29 | 15.49 |  |
|  | **HL-T** | 100.00 |  |  | 105.41 | 10.65 |  | 108.97 | 11.56 | # | 107.14 | 7.03 |  |
|  | **LL-T** | 100.00 |  |  | 104.41 | 15.58 |  | 107.31 | 18.83 | # | 100.39 | 8.05 |  |
|  | **CON** | 100.00 |  |  | 100.89 | 8.87 |  | 97.79 | 6.30 | # | 100.00 | 3.47 |  |
| **Quadriceps MTH** |  | **Ave** | **SD** | **Sig.** | **Ave** | **SD** | **Sig.** | **Ave** | **SD** | **Sig.** | **Ave** | **SD** | **Sig.** |
|  | **BFR-T** | 100.00 |  |  | 103.90 | 4.55 |  | 106.81 | 6.78 | #† | 102.56 | 6.42 | # |
|  | **HL-T** | 100.00 |  |  | 104.65 | 4.62 |  | 114.34 | 15.59 | #†*^*d* | 111.54 | 13.65 | #**d* |
|  | **LL-T** | 100.00 |  |  | 107.00 | 10.27 |  | 109.80 | 9.17 | #†* | 105.91 | 10.56 | # |
|  | **CON ^$^** | 100.00 |  |  | 98.20 | 7.15 | ab | 99.84 | 6.12 | #†*b* | 99.72 | 3.93 | #*b* |
| **Hamstrings MTH** |  | **Ave** | **SD** | **Sig.** | **Ave** | **SD** | **Sig.** | **Ave** | **SD** | **Sig.** | **Ave** | **SD** | **Sig.** |
|  | **BFR-T** | 100.00 |  |  | 105.72 | 13.55 |  | 111.34 | 14.74 | #* | 105.82 | 11.71 | # |
|  | **HL-T** | 100.00 |  |  | 104.86 | 10.07 |  | 115.67 | 22.21 | #*^ | 116.65 | 21.95 | #*^ |
|  | **LL-T** | 100.00 |  |  | 100.70 | 9.17 |  | 107.67 | 11.47 | # | 107.07 | 14.16 | # |
|  | **CON** | 100.00 |  |  | 102.41 | 2.84 |  | 101.70 | 1.77 | # | 103.54 | 1.73 | # |
| **Calf MTH** |  | **Ave** | **SD** | **Sig.** | **Ave** | **SD** | **Sig.** | **Ave** | **SD** | **Sig.** | **Ave** | **SD** | **Sig.** |
|  | **BFR-T** | 100.00 |  |  | 108.66 | 15.21 |  | 112.69 | 14.86 | #* | 109.83 | 15.08 | #* |
|  | **HL-T** | 100.00 |  |  | 103.89 | 11.67 |  | 110.71 | 17.61 | #* | 111.24 | 18.52 | #* |
|  | **LL-T** | 100.00 |  |  | 102.04 | 8.78 |  | 107.16 | 15.57 | # | 104.52 | 13.57 | # |
|  | **CON** | 100.00 |  |  | 98.00 | 4.68 |  | 98.98 | 5.09 | # | 98.88 | 3.89 | # |
| **Tibialis Anterior MTH** |  | **Ave** | **SD** | **Sig.** | **Ave** | **SD** | **Sig.** | **Ave** | **SD** | **Sig.** | **Ave** | **SD** | **Sig.** |
|  | **BFR-T** | 100.00 |  |  | 101.25 | 9.09 |  | 102.79 | 9.27 |  | 102.67 | 7.04 |  |
|  | **HL-T** | 100.00 |  |  | 102.80 | 3.06 |  | 100.87 | 4.44 |  | 101.75 | 4.21 |  |
|  | **LL-T** | 100.00 |  |  | 100.38 | 4.08 |  | 100.26 | 5.38 |  | 99.38 | 3.04 |  |
|  | **CON** | 100.00 |  |  | 100.01 | 2.40 |  | 98.82 | 2.59 |  | 100.79 | 2.65 |  |

* indicates significant difference from Baseline (*P* ≤ 0.05); ^ indicates significant difference from 4 weeks (*P* ≤ 0.05); # main effect for Time vs Baseline (*P* ≤ 0.05); † main effect for Time vs 4 weeks (*P* ≤ 0.05); ‡ main effect for Time vs Week 12 (*P* ≤ 0.05); ^$^ main effect for group vs HL-T (*P* ≤ 0.05); *a* significantly different from BFR-T; *b* significantly different from HL-T*; c* significantly different from LL-T; *d* sig significantly different from CON.
